# Energy transmission using recyclable quantum entanglement

**DOI:** 10.1038/srep30603

**Published:** 2016-07-28

**Authors:** Ming-Yong Ye, Xiu-Min Lin

**Affiliations:** 1 Fujian Provincial Key Laboratory of Quantum Manipulation and New Energy Materials, College of Physics and Energy, Fujian Normal University, Fuzhou 350117, China; 2 Key Laboratory of Quantum Information, University of Science and Technology of China, Chinese Academy of Sciences, Hefei 230026, China; 3 Fujian Provincial Collaborative Innovation Center for Optoelectronic Semiconductors and Efficient Devices, Xiamen, 361005, China

## Abstract

It is known that faster-than-light (FTL) transmission of energy could be achieved if the transmission were considered in the framework of non-relativistic classical mechanics. Here we show that FTL transmission of energy could also be achieved if the transmission were considered in the framework of non-relativistic quantum mechanics. In our transmission protocol a two-spin Heisenberg model is considered and the energy is transmitted by two successive local unitary operations on the initially entangled spins. Our protocol does not mean that FTL transmission can be achieved in reality when the theory of relativity is considered, but it shows that quantum entanglement can be used in a recyclable way in energy transmission.

Faster-than-light (FTL) transmission of information and energy is usually believed to be impossible when the theory of relativity is considered, but in the framework of non-relativistic theory FTL transmission can be achieved. In non-relativistic classical mechanics, a rigid lever is an idealization of a solid lever in which deformation is neglected. As shown in Fig. 1, pressing one side of a rigid lever can lift up the stone on the other side instantaneously. When the position of the stone becomes higher, it must get some energy from the man who pressed the rigid lever. Because the action of the man pressing the lever on one side and the action of the stone going up on the other side happen at the same time, no time is needed to transmit energy. Therefore a rigid lever can be used to transmit energy faster than light. As is known that there is no rigid lever in reality, we must take a step back and only conclude that a lever can be used to transmit energy but the speed can not be faster than light. Here we give a simple example of FTL transmission of energy in the framework of non-relativistic quantum mechanics where two separate spins have a direct interaction. In our energy transmission protocol quantum entanglement plays a role similar to a rigid lever. Quantum entanglement is a very useful resource in quantum information processing^1^. One of its most interesting applications is quantum teleportation, which uses quantum entanglement to transmit quantum state^2^. Motivated by quantum teleportation, Hotta proposes quantum energy teleportation, which uses quantum entanglement to transmit energy^3^,^4^. Our energy transmission protocol is motivated by the minimal quantum energy teleportation^3^,^4^. In both quantum teleportation and quantum energy teleportation, quantum entanglement has been destroyed at the end of the transmission and thus is not recyclable. However our energy transmission protocol gives an example that quantum entanglement can be used in a recyclable way.

## Results

### Energy transmission using recyclable entanglement

**Figure 2:**
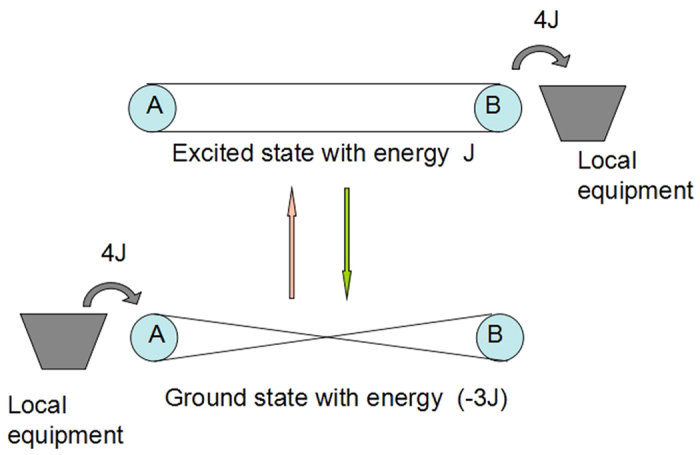
Energy transmission from a local equipment on the sender’s side to a local equipment on the receiver’s side. The sender gives an amount of energy 4J to the system AB and changes the system AB from the ground state |*g*〉 to the excited state |*e*〉. The receiver then takes out an amount of energy 4J from the system AB and changes the system AB from the excited state |*e*〉 back to the ground state |*g*〉.

Consider a system consisting of separate spin A and B with the Heisenberg coupling Hamiltonian


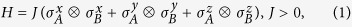


where *σ*^*x*^, *σ*^*y*^ and *σ*^*z*^ are the Pauli operators. The Hamiltonian is widely discussed in spin-based quantum computation^5^,^6^,^7^, where local magnetic field can be used to implement local unitary operations on single spins. The state


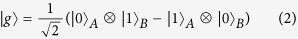


is the ground state of the system with the eigen-energy *E*_*g*_ = −3*J*, and the state


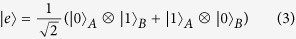


is an excited state of the system with the eigen-energy *E*_*e*_ = *J*, where |0〉 and |1〉 are the eigenstates of *σ*^*z*^ with eigenvalue 1 and −1, respectively. The ground state |*g*〉 and the excited state |*e*〉 can be changed into each other by implementing local unitary operation 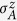 or 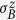. Suppose the system AB is initially in the ground state |*g*〉. The energy transmission from the sender who holds the spin A to the receiver who holds the spin B can be done through the following two steps (see Fig. 2):

1. The sender first performs the unitary operation 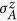 on spin A, which is done by a local equipment on the sender’s side.
2. The receiver then performs the unitary operation 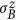 on spin B, which is done by a local equipment on the receiver’s side.

It is assumed that the two local unitary operations are accomplished within a very short time such that the evolution of the system due to the Hamiltonian in Eq. (1) can be ignored when implementing these local operations (see the section of Methods).

In the step 1, the sender’s unitary operation changes the system AB from the ground state |*g*〉 to the excited state |*e*〉, changing the energy of the system AB from *E*_*g*_ to *E*_*e*_. In the step 2, the receiver’s unitary operation changes the system AB from the excited state |*e*〉 back to the ground state |*g*〉 (with an additional global phase *π*), changing the energy of the system AB from *E*_*e*_ back to *E*_*g*_. According to energy conservation, the sender’s local equipment must give an amount of energy 4*J* (i.e., *E*_*e*_ − *E*_*g*_) to the system AB in the step 1, and the receiver’s local equipment must take out an amount of energy 4*J* from the system AB in the step 2. Therefore all the energy that the sender’s local equipment inputs to the system AB has been taken out by the receiver’s local equipment, i.e., an amount of energy 4*J* has been completely transmitted from the sender’s local equipment to the receiver’s local equipment. Note that the system AB turns back to the initial entangled state after the two local unitary operations, so the quantum entanglement has not been destroyed.

## Discussion and Summary

**Figure 3:**
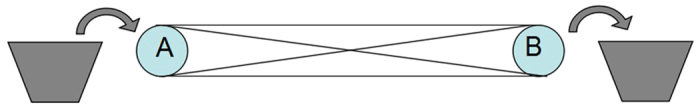
The system AB forms an energy transmission pipe. Energy can be poured into the pipe at one end and taken out from the other end, and the transmission process is repeatable.

The time needed to transmit energy in the above protocol is the sum of the time implementing the sender’s local unitary operation in the step 1, the time implementing the receiver’s local unitary operation in the step 2 and the time interval between them. Since these times can be made arbitrary small in principal (see the section of Methods) and no classical communication is required, energy can be transmitted in any speed and even faster than light. Therefore the protocol is an example that FTL transmission of energy can be achieved in the framework of non-relativistic quantum mechanics. The FTL transmission originates from the fact that there is a direct interaction between the two separate spins. When the theory of relativity is considered, two separate spins can not have a direct interaction. In reality the Heisenberg coupling Hamiltonian is only an approximation, and the time needed to implement the local operations in the protocol can not be made arbitrary small. Therefore similar to the transmission of energy using a lever, we can not conclude from the protocol that FTL transmission of energy can be achieved in reality. The useful thing the protocol reveals is that quantum entanglement can be used in a recyclable way in transmission of energy. In the protocol, the system AB is like an energy transmission pipe (see Fig. 3). Energy can be poured into the pipe at one end and taken out from the other end. However the distance between the two spins with the Heisenberg coupling Hamiltonian can not be large in real quantum system, thus only a very short energy transmission pipe can be obtained. A short energy transmission pipe may be of less practical usage. Hope that the protocol can evoke more discussions about the role of quantum entanglement in energy transmission.

In conclusion, we have shown that two successive local unitary operations on a pair of entangled spins can transmit energy, where the two separate spins have the Heisenberg coupling Hamiltonian. The special entanglement we used ensures that the effect of a unitary operation on one spin can be eliminated by a unitary operation on the other, which leads the energy transmission process to be repeatable. The protocol is an example of FTL transmission in the framework of non-relativistic quantum mechanics, which does not mean that FTL transmission can be achieved in reality. The meaningful thing showed by the protocol is that quantum entanglement can be used in a recyclable way in the transmission of energy.

## Methods

The local unitary operations in the steps 1 and 2 of the transmission can be done within an arbitrary small time in the following ways. Assume the energy transmission process starts at *t* = 0. To implement the local unitary operation 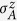 in the step 1 the sender can add the local term


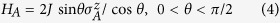


to the system Hamiltonian from *t* = 0 to *t* = *T*_*A*_ = *πħ*cos *θ*/(4*J*) by applying a local magnetic field on spin A just like in the model of spin-based quantum computation^5^. During the time interval *t* ∈ [0, *T*_*A*_], the system Hamiltonian is


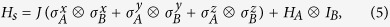


where *I* is the identity operator. When the local term *H*_*A*_ is very large, i.e., *θ* is very close to *π*/2, *T*_*A*_ will be very close to 0 and the time evolution operator will be


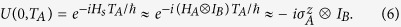


That is to say there is *T*_*A*_ → 0 and 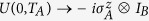, which means the sender can implement an arbitrary good local unitary operation 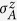 (with a global phase) in an arbitrary small time *T*_*A*_ in principle if she uses an arbitrary large local term *H*_*A*_. The similar result can be obtained for the receiver’s local unitary operation.

## Additional Information

**How to cite this article**: Ye, M.-Y. and Lin, X.-M. Energy transmission using recyclable quantum entanglement. *Sci. Rep.* **6**, 30603; doi: 10.1038/srep30603 (2016).

## Figures and Tables

**Figure 1:**
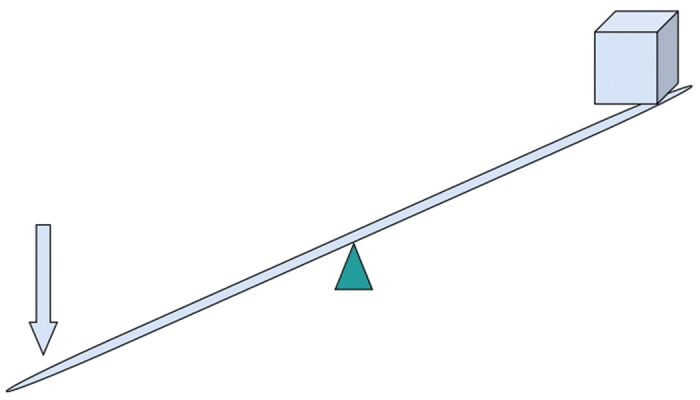
Energy transmission by a rigid lever. Pressing one side of the lever can lift up the stone on the other side that makes the stone have a higher energy.
